# Evidence of partner similarity for autistic traits, systemizing, and theory of mind via facial expressions

**DOI:** 10.1038/s41598-022-11592-z

**Published:** 2022-05-19

**Authors:** Gareth Richards, Simon Baron-Cohen, Varun Warrier, Ben Mellor, Jessica Davies, Laura Gee, John Galvin

**Affiliations:** 1 School of Psychology, Faculty of Medical Sciences, Newcastle University, 4.32 Dame Margaret Barbour Building, Wallace Street, Newcastle upon Tyne, NE2 4DR UK; 2 Autism Research Centre, Department of Psychiatry, University of Cambridge, Cambridge, UK; 3 Department of Psychology, Birmingham City University, Birmingham, UK

**Keywords:** Human behaviour, Psychology

## Abstract

It has been hypothesised that romantic partners are more similar than chance in relation to autistic traits. To test this theory, we recruited n = 105 heterosexual couples and examined within-couple correlations for autistic traits [measured using the Autism Spectrum Quotient (AQ)], empathizing [measured using the Empathy Quotient (EQ)], and systemizing [measured using the Systemizing Quotient-Revised (SQ-R)]. For a subsample that attended the lab (n = 58 couples), we also investigated theory of mind via facial expressions using the Reading the Mind in the Eyes Test (RMET) and attention to detail, a component within systemizing, using the Embedded Figures Task (EFT). Variable-centred analyses revealed positive within-couple correlations for all measures except EQ, although these effects were only statistically significant for unmarried couples and not for married/engaged couples. Follow-up analyses indicated that the observed couple similarity effects are likely consistent with people pairing with those more similar than chance (initial assortment) rather than becoming alike over time (convergence), and to seeking out self-resembling partners (active assortment) rather than pairing in this manner via social stratification processes (social homogamy). Additionally, a significant within-couple correlation for autistic traits was observed at the meta-analytic level. However, it should be noted that the meta-analytic effect size estimate was small (*r* = 0.153) and indicates that only ~ 2% of variance in a person’s score on a phenotypic measure of autistic traits can be predicted by that of their partner.

## Introduction

Autism is characterised by unusually routine behaviours, narrow interests, sensory hyper-sensitivity, social and communicative problems, and difficulties in adjusting to unexpected change^1^. There is a marked sex difference in autism diagnosis, with approximately 3–4 males being diagnosed per every 1 female^2–4^, an effect that may in part be explained in terms of biological factors such as elevated foetal sex steroid exposure^5–8^ and social factors such as gender stereotyping^4,9–11^. Although often considered purely categorically in terms of diagnosis, personality and behavioural characteristics related to autism (henceforth ‘autistic traits’) can be measured quantitatively and are continuously distributed throughout the general population^12–14^.

While autistic people on average are less likely to marry or be in long-term relationships^15,16^, many have partners^17^ and relatively few report not being interested in being in a romantic relationship^18^. The assortative mating theory hypothesised that autism could be subject to positive assortative mating^19–23^. Essentially, this predicts that autistic individuals are more likely than chance to form romantic relationships with other autistic people. A similar process may also occur throughout the general population, whereby a person is more likely than chance to partner with someone who has a similar level of autistic traits to themself. However, this phenomenon is not unique to autism or autistic traits, and assortative mating can occur for a multitude of variables including aspects of demography, attitudes and values, abilities and intelligence, mental health and wellbeing, habitual behaviours and lifestyle factors, personality, and physical and physiological characteristics^24–32^. There is also more than one process by which assortment could operate. For instance, it may be that individuals with similar levels of a given trait consciously or unconsciously seek each other out as romantic partners (active assortment) or that individuals with similar levels of that trait are more likely than chance to share other characteristics, such as a working environment, which may lead to an increased likelihood of a relationship starting (social homogamy); in addition, individuals may begin relationships with others who are more similar to themselves than expected by chance (initial assortment) or become more similar to their partner over the course of their relationship (convergence)^24,29^. Assortment also occurs in non-romantic relationships, with friends being more alike than chance for many variables^33,34^, including autistic traits^35^.

Evidence for autism being subject to assortative mating comes from a study by Nordsletten et al.^36^ which reported that a person diagnosed with autism is 10–12 times more likely to marry or have a child with another autistic person than is someone without such a diagnosis. However, due to matching five controls to every case, the partner correlation (*r* = 0.47) observed by Nordsletten et al.^36^ has been estimated to reflect a smaller effect (~ *r* = 0.28) in the general population^37^. Although a study by Yengo et al.^38^, which used genome wide association study (GWAS) data, did not observe a statistically signficant genotypic correlation after correcting for multiple testing, a more recently published study by Connolly et al.^39^ reported greater genetic similarity than expected by chance in the parents of autistic children. Additionally, several studies have reported positive within-couple correlations for autistic traits^39–45^, though others have reported null^46–49^ or ambiguous findings^50^.

Previous studies have examined autism as one entity and autistic traits as one set of traits. Here we consider autistic traits as a whole and as a combination of different traits. With excellent attention to detail, and the ability to remain focussed on pattern-based tasks, autistic people are more likely to work in Science, Technology, Engineering, and Mathematics (STEM)^12,51–54^. This effect extends to people who display higher than average levels of autistic traits but do not have a clinical diagnosis^12,55–59^. Empathizing and systemizing may therefore be important variables to consider within the assortative mating paradigm. Empathizing is the ability to recognise mental states in other people and to respond to those mental states with an appropriate emotion. Systemizing is the ability to analyse or build systems based on input-operation-output (if-and-then) rules. Females on average score higher than males for self-reported empathizing, whereas males on average score higher than females on measures of systemizing. Autistic people on average score higher on systemizing and lower on empathizing than non-autistic people^60^. Some research has used behavioural measures to investigate these constructs, such as the Reading the Mind in the Eyes Test (RMET)^61^, an advanced test of theory of mind^62^, and the Embedded Figures Task (EFT)^63^, a test of attention to detail, necessary for systemizing. The RMET and EFT show the same performance patterns as self-report measures: more specifically, on the RMET, typical females > typical males > autistic people^62^, whereas on the EFT, autistic people > typical males > typical females^55,64^. Interestingly there is no published research examining within-couple correlations for measures of systemizing or empathizing, although there is evidence of assortative mating for related constructs such as cognitive complexity^65^ and emotional intelligence^26^.

The current study aims to increase our understanding of the processes that might underpin assortative mating as it relates to autism. More specifically, we examined within-couple correlations for quantitative self-report measures of autistic traits (the Autism Spectrum Quotient or AQ^12^), empathizing (the Empathy Quotient or EQ^66^), and systemizing (the Systemizing Quotient-Revised or SQ-R^67^), as well as the standardised difference between empathizing and systemizing (D score). Additionally, we investigated behavioural measures that map onto empathizing (the Reading the Mind in the Eyes Test or RMET^61^) and systemizing (the Embedded Figures Test or EFT^63^). We predicted that: (1) average sex differences would be observed for each of these measures (males > females for AQ, SQ-R, D score, and EFT; females > males for EQ and RMET); (2) variables associated with social homogamy (age, educational attainment, and STEM status) would correlate positively within couples; (3) autistic traits would be positively correlated within couples; (4) within-couple correlations for autistic traits would reflect initial assortment rather than convergence; (5) within-couple similarity for autistic traits might reflect social homogamy for age and educational attainment or active assortment (no directional prediction was made here); and (6) within-couple correlations for autistic traits would be stronger in couples in which both partners worked/studied in STEM.

## Results

### Demographic information, intercorrelations, and sex differences

Both male and female data were available for 105 couples. Most participants were self-reported White/Caucasian (males: n = 95, 90.48%; females: n = 95, 91.35% [one missing datapoint]) employed (males: n = 86, 81.90%; females: n = 89, 84.76%), and relatively few were currently students (males: n = 18, 17.14%; females, n = 27, 25.71%). The levels of educational attainment reported are as follows: no qualifications (males, n = 4, 3.81%; females, n = 1, 0.95%), GCSE or equivalent (males: n = 21, 20.00%; females, n = 15, 14.29%), A level or equivalent (males: n = 27, 25.71%; females: n = 35, 33.33%), Bachelor’s Degree (males: n = 38, 36.19%; females: n = 32, 30.48%), Master’s Degree (males: n = 8, 7.62%; females: n = 11, 10.48%), Doctorate Degree (males: n = 7, 6.67%; females: n = 11, 10.48%). One male (0.95%) and no females (0.00%) disclosed having an autism diagnosis, though 8 males (7.69%) and 3 females (2.86% [one missing datapoint]) suspected they might be autistic. Most participants reported living with their partner, although there was a slight discrepancy in that fewer males than females reported this (males: n = 73, 69.52%; females: n = 76, 72.38%). For marital status, there was no such discrepancy: 59 couples (56.19%) were not married, 8 (7.62%) were engaged, and 38 (36.19%) were married.

**Figure 1:**
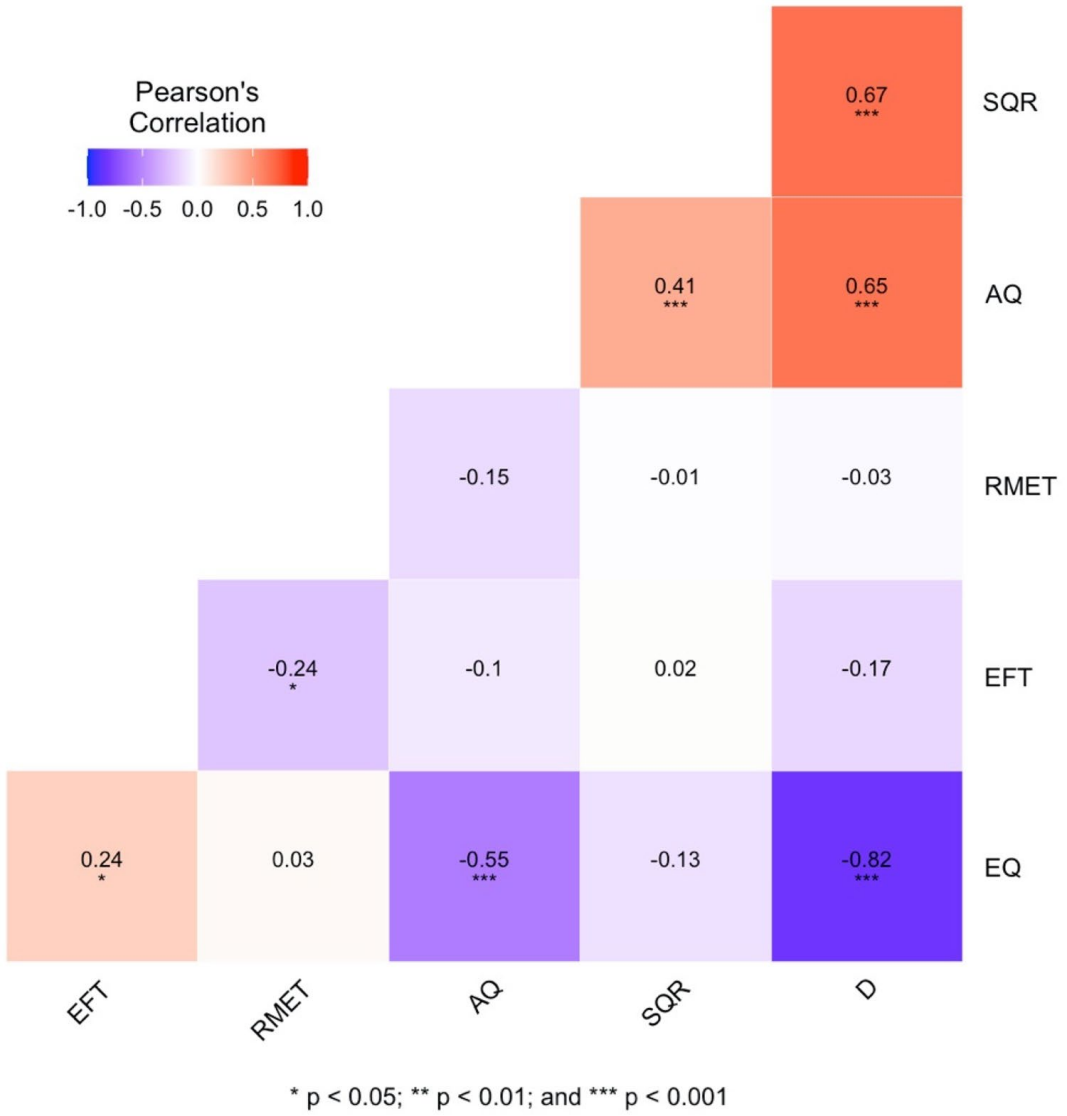
Correlation heatmap for autism-related variables.

Although not specified as part of our pre-registration, intercorrelations between the autism-related variables are depicted in Fig. 1. As might be expected, the strongest correlations were between D and each of the variables from which it is derived, namely EQ (*r* = − 0.82) and SQ-R (*r* = 0.67). Notably, AQ scores correlated negatively with EQ (*r* = − 0.55) and positively with SQ-R (*r* = 0.41), as previously reported^67^. As predicted, males on average scored higher than their female partners on AQ, SQ-R, and D score, and on average achieved faster times on the EFT (Table 1).

**Table 1:** Descriptive statistics and sex differences for autism-related variables.

	Male			Female			Difference			
	*n*	*M*	*SD*	*n*	*M*	*SD*	*t*	*df*	*p*	*d*
AQ	104	18.01	7.43	104	15.30	6.65	3.323	103	**0.001**	**0.384**
EQ	102	40.48	13.01	102	52.68	12.99	− 6.641	101	**< 0.001**	− **0.938**
SQ-R	103	60.16	21.50	103	46.04	17.16	6.040	102	**< 0.001**	**0.723**
D	101	0.039	0.102	101	− 0.086	0.103	9.662	100	**< 0.001**	**1.220**
RMET	55	23.85	4.96	55	24.84	4.95	− 1.386	54	0.172	− 0.198
EFT	58	32.24	21.24	58	41.34	27.60	− 2.587	57	**0.012**	− **0.365**

AQ, Autism Spectrum Quotient; EQ, Empathy Quotient; SQ-R, Systemizing Quotient-Revised; D, difference in standardized EQ (E) and SQ-R (S) scores; RMET, Reading the Mind in the Eyes Test; EFT, Embedded Figures Task. Effects in bold are statistically significant (*p* < 0.05, two-tailed).

### Within-couple correlations for social homogamy variables

Partners’ ages were strongly positively correlated, *r*(103) = 0.964, *p* < 0.001. A Spearman’s test also demonstrated that partners’ levels of educational attainment were positively correlated, *r*_s_(103) = 0.399, *p* < 0.001, and a chi-square test showed that those studying/working in STEM were more likely than chance to have a partner who was also studying/working in STEM, χ^2^ (1, 87) = 11.481, *p* < 0.001, φ = − 0.39.

### Variable-centred analysis of autistic traits

**Figure 2:**
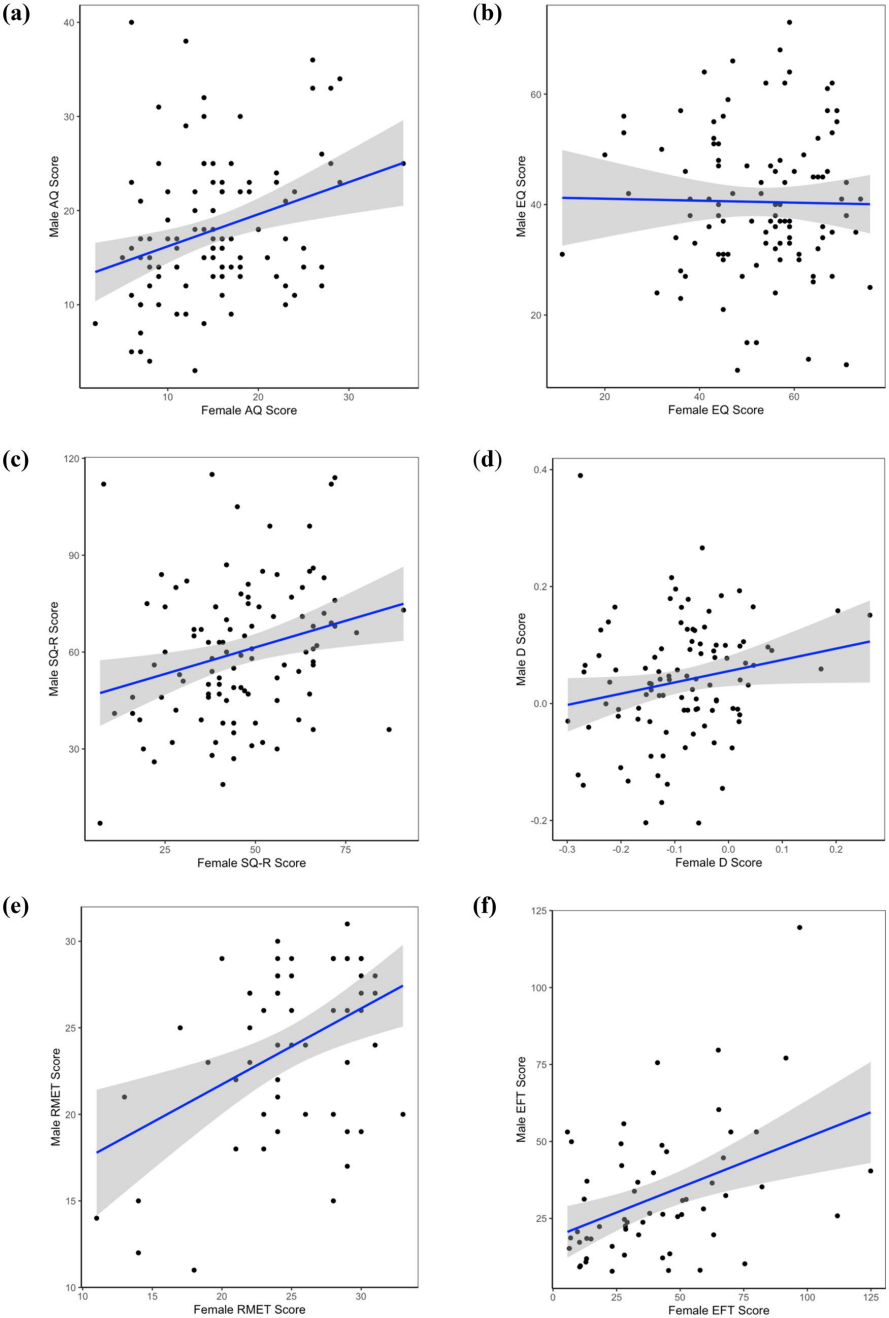
Scatterplots with regression lines and 95% confidence intervals demonstrating within-couple correlations observed for AQ (**a**), EQ (**b**), SQ-R (**c**), D score (**d**), RMET (**e**), and EFT (**f**).

Although we preregistered an analysis based on Pearson’s correlations, some of the scale distributions were non-normal, and Spearman’s correlations were used instead. Six Spearman’s tests (Bonferroni-corrected required α level: *p* < 0.008) were conducted to test for within-couple correlations for the autism-related variables (for scatterplots, see Fig. 2). After Bonferroni correction, significant positive correlations were observed for AQ (*r*_*s*_[102] = 0.280, *p* = 0.004), SQ-R (*r*_s_[101] = 0.279, *p* = 0.004), and EFT (*r*_s_[56] = 0.373, *p* = 0.004). The correlations for D score and RMET were nominally significant but did not survive Bonferroni correction: D score, *r*_s_(99) = 0.233, *p* = 0.019, RMET, *r*_s_(53) = 0.329, *p* = 0.014. No correlation was observed for EQ (*r*_s_[100]  = − 0.004, *p* = 0.969).

At the suggestion of a reviewer, we stratified the above analyses by marital status (unmarried or married/engaged). All outcome variables other than EQ exhibited statistically significant positive within-couple correlations for unmarried couples, whereas no significant correlations were observed for the married/engaged couples. We then used Fisher’s *r*-to-*z* tests to compare the strength of correlation across the two groups. These revealed that the within-couple correlation for AQ was significantly stronger in unmarried couples, and a similar effect was observed for SQ-R though it did not reach statistical significance (Table 2). However, no effects observed here in relation to the Fisher's *r*-to-*z* tests would survive Bonferroni correction.

**Table 2:** Within-couple correlations for autism-related variables stratified by marital status.

	Unmarried couples			Married/engaged couples			Difference	
	*n*	*r*_s_	*p*	*n*	*r*_s_	*p*	*z*	*p*
AQ	59	**0.434**	**0.001**	45	0.058	0.704	**1.99**	**0.047**
EQ	59	0.095	0.476	43	− 0.106	0.497	0.97	0.332
SQ-R	58	**0.446**	**< 0.001**	45	0.083	0.588	1.93	0.054
D	58	**0.360**	**0.005**	43	0.104	0.507	1.31	0.190
RMET	35	**0.487**	**0.003**	20	0.046	0.847	1.62	0.105
EFT	37	**0.518**	**0.001**	21	0.196	0.394	1.29	0.197

AQ, Autism Spectrum Quotient; EQ, Empathy Quotient; SQ-R, Systemizing Quotient-Revised; D, difference in standardized EQ (E) and SQ-R (S) scores; RMET, Reading the Mind in the Eyes Test; EFT, Embedded Figures Task. ﻿Effects in bold are statistically significant at the conventional threshold of *p* < 0.05 (two-tailed).

### Initial assortment versus convergence

To investigate whether the within-couple correlations for autism-related variables were explainable by couples being more similar than chance to begin with (initial assortment) or becoming more alike throughout the course of their relationship (convergence), we correlated the sex-standardised within-couple difference scores for autism-related variables with length of relationship. Essentially, if length or relationship is correlated with the difference score, it suggests that partners may become more similar (negative correlation) or more dissimilar (positive correlation) over the course of their relationship, and so provides evidence against initial assortment^29^. As both males and females reported the length of their relationship, we correlated these to check for similarity (*r*[102] = 0.999, *p* < 0.001) before averaging the two measurements for use in further analyses. The averaged measure showed a wide range of relationship lengths from 1 month to 60 years (*M* = 9.37, *SD* = 11.03).

Our pre-registration specified that we would use Pearson’s correlations for this analysis. However, we used linear regression instead, as this allowed for inclusion of marital status (unmarried vs. married/engaged) as a covariate. Relationship length was not associated with the within-couple differences for AQ, *b*(101) = − 0.008, *p* = 0.313, EQ, *b*(99) = − 0.002, *p* = 0.816, SQ-R, *b*(100) = − 0.009, *p* = 0.238, D, *b*(98) = − 0.003, *p* = 0.721, RMET, *b*(52) = 0.005, *p* = 0.581, or EFT, *b*(55) = − 0.003, *p* = 0.717. These findings therefore indicate that within-couple correlations for autism-related variables are more likely attributable to initial assortment than convergence.

### Active assortment versus social homogamy

We next aimed to investigate whether within-couple correlations for autism-related variables may be better explained by individuals consciously or unconsciously seeking out similar partners (active assortment) or by people tending to pair with others around them who, on average, are more similar than chance due to social stratification effects (social homogamy). We first correlated the within-couple sex-standardised difference scores for the autism-related variables with the couples’ absolute (i.e., unsigned) difference in age. The idea is that if similarity for age is positively correlated with similarity for an autism-related variable, then the within-couple correlation for that autism-related variable may be explained by social homogamy effects. Although we pre-registered to use Pearson’s correlations, we used linear regression analyses instead, as this allowed for marital status (unmarried or married/engaged) to be included as a covariate. We observed no correlation between absolute age difference and within-couple differences for AQ (*b*[101] = − 0.003, *p* = 0.922), SQ-R (*b*[100] = 0.006, *p* = 0.835), D (*b*[98] = − 0.051, *p* = 0.089), RMET (*b*[52] = 0.047, *p* = 0.106), or EFT (*b*[55] = 0.004, *p* = 0.913). However, there was a significant correlation for EQ, *b*(99) = − 0.070, *p* = 0.014. This effect suggests that couples with disparate age-gaps may be more similar in empathizing than expected by chance.

We then examined whether there were correlations between couple similarity for level of educational attainment and autism-related variables. We initially pre-registered Spearman’s correlations for this analysis, though again adopted the approach of using linear regression to control for marital status as a covariate. Couple similarity for educational attainment was not significantly associated with couple similarity for AQ (*b*[101] = 0.035, *p* = 0.680), EQ (*b*[99] = − 0.043, *p* = 0.649), SQ-R (*b*[100] = − 0.005, *p* = 0.950), D score (*b*[98] = − 0.071, *p* = 0.414), RMET (*b*[52] = 0.220, *p* = 0.065), or EFT (*b*[55] = 0.165, *p* = 0.212).

Finally, we examined whether couples who study and/or work in STEM are more similar for autism-related variables than are couples for whom either one member or both members do not study or work in STEM. Of those participants who reported current employment and/or student status, there were 35 males and 33 females who worked or studied in STEM areas, and 57 males and 61 females who did not study or work in STEM areas. We compared the 20 couples for whom both members were in STEM with the 67 couples for whom either one or neither member was in STEM. These groups of couples did not differ in their levels of similarity on the AQ (*t*[37.315] = − 0.464, *p* = 0.646), EQ (*t*[45.323] = 0.304, *p* = 0.762), SQ-R (*t*[37.327] = − 0.072, *p* = 0.943), D score (*t*[36.017] = 0.077, *p* = 0.939), RMET (*t*[9.925] = 0.269, *p* = 0.793), or EFT (*t*[12.736] = − 0.197, *p* = 0.847) (equal variances were not assumed in each case). The overall pattern of results observed is therefore consistent with active assortment. However, as we only investigated associations between similarity in autism-related variables and similarity in age, educational attainment, and STEM status, the influence of other aspects of social homogamy (e.g., similarity in religious beliefs) cannot be ruled out.

### Couple-centred analysis of autistic traits

We next aimed to determine whether a participant’s scores for autism-related variables were more similar (i.e., numerically closer) to those of their partner than would be expected by chance. To do this, we first calculated sex-standardised absolute (unsigned) difference scores for each of the relevant variables. Next, we calculated the difference scores between any given male and all females that were not his partner (and, therefore, any given female and all males that were not her partner) and took the average. We then used six paired-samples *t* tests (Bonferroni-corrected required α level: *p* < 0.008) to compare actual couples’ difference scores with those of random pairings. These determined that the AQ, SQ-R, RMET and EFT difference scores for actual couples were smaller (i.e., more similar) than those calculated from random pairings, and that the observed effect sizes were small^68^; no significant effects were observed for EQ and D score (Table 3).

**Table 3:** Comparison of actual couple similarity for autism-related variables with the average difference scores calculated by pairing each participant with all other potential other-sex partners in the dataset.

	Actual couple difference		Average difference of all other pairings		Difference			
	*M*	*SD*	*M*	*SD*	*t*	*df*	*p*	*d*
AQ	0.891	0.770	1.118	0.433	− 3.227	103	**0.002**	− **0.346**
EQ	1.109	0.861	1.111	0.384	− 0.025	101	0.980	− 0.003
SQ-R	0.928	0.779	1.128	0.410	− 2.761	102	**0.007**	**− 0.305**
D	0.973	0.788	1.092	0.433	− 1.817	100	0.072	− 0.170
RMET	0.837	0.634	1.101	0.408	− 2.817	54	**0.007**	**− 0.491**
EFT	0.788	0.722	1.071	0.520	− 2.847	57	**0.006**	**− 0.443**

AQ, Autism Spectrum Quotient; EQ, Empathy Quotient; SQ-R, Systemizing Quotient-Revised; D, difference in standardized EQ (E) and SQ-R (S) scores; RMET, Reading the Mind in the Eyes Test; EFT, Embedded Figures Task. Effects in bold are statistically significant (*p* < 0.05, two-tailed) and remained so after Bonferroni correction (required α level: *p* < 0.008).

### Random-effects meta-analysis of the within-couple correlation for autistic traits

**Figure 3:**
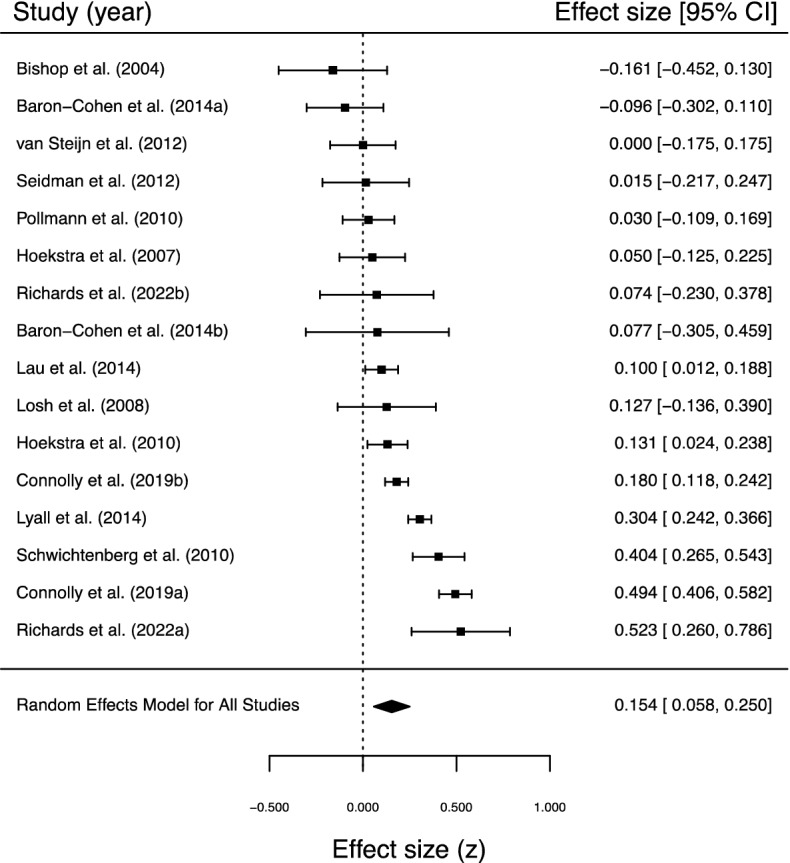
Forest plot for within-couple correlations for autistic traits. Note Baron-Cohen et al. (2014a) = parents of autistic children; Baron-Cohen et al. (2014b) = parents without autistic children; Connolly et al. (2019a) = Autism Genome Project sample; Connolly et al. (2019b) = Simons Simplex Collection sample; Richards et al. (2022a) = unmarried sample; Richards et al. (2022b) = married/engaged sample.

**Figure 4:**
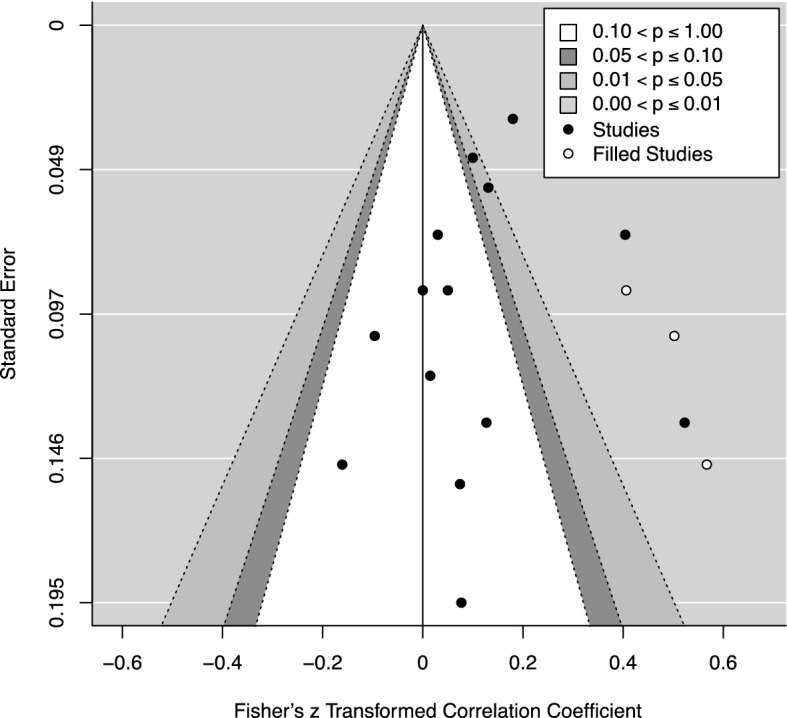
Contour-enhanced funnel plot for studies for which within-couple correlations for autistic traits measures could be determined. Black circles represent individual studies; white circles represent the hypothesised missing studies imputed via the trim and fill procedure.

Considering the exploratory nature of this analysis, we included all samples identified for which a within-couple correlation for an autistic traits measure was reported, as well as those for which the within-couple correlation could be calculated from available data (16 samples from 13 studies; see Table 4). The meta-analysis (k = 16, n = 5444; see Fig. 3 for forest plot) returned a statistically significant positive correlation, *r* = 0.153 (95% CI 0.058, 0.245) [*z* = 0.154; 95% CI 0.058, 0.250], *p* = 0.002, and significant heterogeneity was observed, Q (15) = 105.303, *p* < 0.0001, τ^2^ = 0.029, *I*^2^ = 87.97%. Removal of any one sample did not change these results noticeably. Egger’s regression test^69^ was not significant, *z* = − 1.433, *p* = 0.152, suggesting absence of publication bias. However, the trim and fill procedure^70^ estimated the presence of three missing studies on the right side (for contour-enhanced funnel plot, see Fig. 4). Imputing these hypothesised studies resulted in a model that was not qualitatively different from the original: *r* = 0.198 (95% CI 0.105, 0.289) [*z* = 0.201; 95% CI 0.105, 0.298], *p* < 0.0001; heterogeneity, Q (18) = 121.943, *p* < 0.0001, τ^2^ = 0.036, *I*^2^ = 89.02%.

Due to the high level of heterogeneity observed (*I*^2^: 25% = low; 50% = moderate; 75% = high^71^), and because the studies included in the meta-analysis appeared to differ considerably, we investigated the potential influence of type of sample via a moderator analysis. More specifically, we partitioned the samples into one of three groups: (1) couples who were parents of autistic children (k = 8), (2) couples who were not parents of autistic children (including those without children) (k = 5), and mixed samples (those which included both parents who had autistic children and parents who did not have autistic children) (k = 3). Subgroup analysis revealed positive correlations in each group, though none reached statistical significance at the *p* < 0.05 level: mixed sample, *r* = 0.222 (95% CI − 0.001, 0.424) [*z* = 0.226; 95% CI − 0.001, 0.452], *p* = 0.051; parents of autistic children, *r* = 0.120 (95% CI − 0.017, 0.253) [*z* = 0.121; 95% CI − 0.017, 0.259], *p* = 0.085; couples who were not parents of autistic children, *r* = 0.161 (95% CI − 0.028, 0.338) [*z* = 0.162; 95% CI − 0.028, 0.352], *p* = 0.095. The formal test of moderation was not statistically significant, Q_M_ (2) = 0.605, *p* = 0.739.

**Table 4:** Overview of studies included in the meta-analysis of within-couple correlations for autistic traits.

References	Year	Journal	Location	Population	Measurement	*n*	*Effect size*	*p*
Bishop et al.^72^	2004	*Journal of Child Psychology and Psychiatry*	Australia	Parents of autistic children (Western Australia Family Study of Autistic Spectrum Disorders) and control parents (recruited via advertisements sent to schools in the Perth Metropolitan Region)	Presence/absence of Broader Autism Phenotype (determined via a principal components analysis of the AQ Social Skill and Communication subscales)	49	ϕ = − 0.16	0.459
Constantino and Todd^40^	2005	*Biological Psychiatry*	USA	Missouri Twin Study (parents of twins)	SRS	285	*ICC* = 0.38	< 0.05
Hoekstra et al.^46^	2007	*Archives of Pediatrics & Adolescent Medicine*	Netherlands	Parents of twins	Dutch AQ	128	*r* = 0.05	0.59
Losh et al.^47^	2008	*American Journal of Medical Genetics, Part B: Neuropsychiatric Genetics*	USA	Parents of autistic children (simplex families, n = 35; multiplex families, n = 23)	Factor 1: Language Factor 2: Rigidity Factor 3: Anxiety Factor 4: Sociability	58 58 58 58	*r*_*s*_ = 0.01–0.23 *r*_*s*_ = 0.01–0.23 *r*_*s*_ = 0.01–0.23 *r*_*s*_ = 0.01–0.23	n.s n.s n.s n.s
Virkud et al.^45^	2009	*American Journal of Medical Genetics, Part B: Neuropsychiatric Genetics*	USA	Autism Genetic Resource Exchange (parents of autistic children [multiplex families]); parents of Washington University male siblings study (simplex families)	SRS	99	*ICC* = 0.26	< 0.01
Hoekstra et al.^42^	2010	*Behavior Genetics*	Netherlands	Parents of twins	Dutch AQ-Short	305	*r* = 0.13	< 0.05?
Schwichtenburg et al.^44^	2010	*Journal of Child Psychology and Psychiatry*	USA	Parents sampled by UCD (n = 115) and UCLA (n = 102) of at least one autistic child n = 135 or no autistic (but typically developing) child n = 82	SRS	217	β = 0.34	< 0.05?
Pollmann et al.^48^	2010	*Journal of Autism and Developmental Disorders*	Netherlands	Couples married for 10 months	Modified Dutch AQ-Short	195	*r* = 0.03	n.s
Seidman et al.^50^	2012	*Journal of Autism and Developmental Disorders*	Israel	Parents of autistic children	BAPQ Total (Hebrew) (SR + IR) BAPQ Aloof (Hebrew) (SR + IR) BAPQ Rigid (Hebrew) (SR + IR) BAPQ Pragmatic Language (Hebrew) (SR + IR)	76 76 76 76	– *r* = -0.33 – *r* = 0.36	– 0.04 – 0.03
van Steijn et al.^49^	2012	*Journal of Child Psychology and Psychiatry*	Netherlands	Parents of autistic children (with or without ADHD)	AQ (alternative scoring method)	121	*r* = 0.0	n.s
Baron-Cohen et al.^14^	2014	*PLoS ONE*	UK	Parents of autistic children in the Cambridge Autism Research Database (CARD)	AQ	92	*r* = − 0.096	0.365
			UK	Parents without autistic children in the Cambridge Autism Research Database (CARD)	AQ	29	*r* = 0.077	0.693
Lau et al.^73^	2014	*Journal of Autism and Developmental Disorders*	Taiwan	Parents of autistic children	AQ-Chinese	491	ϕ = 0.10	0.032
Lyall et al.^43^	2014	*JAMA Psychiatry*	USA	Nurses’ Health Study II parents of autistic children	SRS	1198*	*r* = 0.25	-
			USA	Nurses’ Health Study II control parents	SRS	1198*	*r* = 0.34	-
Duvekot et al.^41^	2016	*Journal of Child Psychology and Psychiatry*	Netherlands	Social Spectrum Study parents of children referred for various mental health conditions; 159 families of children at risk for autism (parent-reported SRS-2 total score of ≥ 75) and 72 families of children considered not at risk for autism (parent-reported SRS-2 total score of < 75)	SRS-2	231	*b* = 0.27	< 0.001
Connolly et al.^39^	2019	*Biological Psychiatry*	Europe/North America	Autism Genome Project (parents of autistic children [simplex and multiplex families])	BAPQ Total (SR + IR) BAPQ Aloof (SR + IR) BAPQ Rigid (SR + IR) BAPQ Pragmatic Language (SR + IR) SRS-A Total (IR) SRS-A Awareness (IR) SRS-A Cognition (IR) SRS-A Mannerisms (IR) SRS-A Motivation (IR) SRS-A Communication (IR)	270 270 270 270 428 428 428 428 428 428	*r* = 0.37 – *r* = 0.28 *r* = 0.43 *r*_s_ = 0.44 *r*_s_ = 0.31 *r*_s_ = 0.45 *r*_s_ = 0.42 – *r*_s_ = 0.40	< 0.001 n.s < 0.001 < 0.001 < 0.001 < 0.001 < 0.001 < 0.001 n.s < 0.001
			North America	Simons Simplex Collection (parents of autistic children [simplex families])	BAPQ Total (SR) BAPQ Aloof (SR) BAPQ Rigid (SR) BAPQ Pragmatic Language (SR) SRS-A Total (IR) SRS-A Awareness (IR) SRS-A Cognition (IR) SRS-A Mannerisms (IR) SRS-A Motivation (IR) SRS-A Communication (IR)	1945 1945 1945 1945 1953 1953 1953 1953 1953 1953	– – – – *r*_s_ = 0.34 - *r*_s_ = 0.32 *r*_s_ = 0.29 - *r*_s_ = 0.30	n.s n.s n.s n.s < 0.001 n.s < 0.001 < 0.001 n.s < 0.001
Richards et al. (current study)	2022	*Scientific Reports*	UK	Unmarried heterosexual couples from the general population	AQ	59	*r*_s_ = 0.434	0.001
			UK	Married/engaged heterosexual couples from the general population	AQ	45	*r*_s_ = 0.058	0.704

AQ, Autism Spectrum Quotient; BAPQ, Broad Autism Phenotype Questionnaire; SRS, Social Responsiveness Scale. *The two samples of Lyall et al. were analysed together; n = 1198 couples were included, though it is unclear how many were parents of autistic children and how many were control parents.

## Discussion

The current study aimed to test whether autistic traits are correlated within heterosexual couples in the general population. The main finding was that quantitative measures of autistic traits (AQ), systemizing (SQ-R), empathizing relative to systemizing (D score), the ability to read emotions in the eye region (RMET), and spatial skills (EFT) (but not empathizing; EQ) were all positively correlated, albeit the effects observed for D and RMET were no longer statistically significant after Bonferroni correction. Interestingly, statistically significant effects were observed for the subsample of unmarried couples, but not for the subsample that was either married or engaged. The couple-similarity effects also appear to be better explained by active assortment than social homogamy, and by initial assortment rather than convergence. Additionally, when we compared within-couple sex-standardised absolute (i.e., unsigned) difference scores for these variables with sex-standardised absolute difference scores calculated as the average of all other possible male–female pairings within the dataset, we found that actual couples were more similar for AQ, SQ-R, RMET, and EFT than would be expected under the assumption of random mating. We also observed a small but statistically significant within-couple correlation for autistic traits in a meta-analysis of 16 samples (including the current study).

A number of investigations have examined within-couple correlations for autistic trait variables, the majority of which have focused on very specific samples such as the parents of autistic children^39,43–45,47,49,50,73^, parents of twins^40,42,46^, and parents of children with various mental health conditions^41^. Although two of these studies have also included samples of parents of typically developing children^43,44^ and another reported on a sample of newlyweds^48^, the nature of these effects within the general population remains relatively unexplored. A novel aspect of the current study is that we demonstrated statistically significant positive within-couple correlations (and increased within-couple similarity) for self-reported systemizing (SQ-R scores) as well as for a behavioural/cognitive skill that likely underpins systemizing ability (speed on the EFT). This is noteworthy considering that systemizing is the trait upon which assortative mating in relation to autism was initially hypothesised to act^20–22^. However, SQ-R scores and speed on the EFT were uncorrelated, a finding which questions the idea that the latter is a behavioural measure of systemizing.

It remains unclear why autistic traits and systemizing are correlated within couples. It may also be a mistake to conflate higher levels of autistic traits with a generalised deficit in social communication skills^74^, as different levels of autistic traits may simply reflect different styles of social communication, cognition, and behaviour. Evidence to support this idea comes from studies showing that information transfer is more effective between autistic adults than it is between autistic and non-autistic adults^75^, and that autistic people tend to show a preference for interactions with other autistic (rather than non-autistic) people^75–77^. These observations may provide a basis for explaining why assortment occurs for autistic traits and systemizing, i.e., because those with high levels of these traits tend to communicate with each other more effectively, increasing the chances of a relationship developing. Equally, as the current study detected within-couple correlations across a range of distinct albeit theoretically related measures, the findings might simply reflect the generalised tendency for couples to be more alike than different^24,30^. It is surprising, however, that we did not observe significant results for empathizing because effects consistent with assortative mating have been reported for the closely related construct of emotional intelligence^26^ (although see also^78^). It is also notable that there was no significant correlation between the self-report empathizing measure (EQ) and the behavioural theory of mind via facial expressions task (RMET). Although speculative, the fact that the latter showed assortment effects whereas the former did not might suggest that self-report measures of empathy introduce social desirability. This is because one could dishonestly manipulate their answers to a self-report measure such as the EQ, to achieve a high score, whereas this is not possible for a behavioural measure such as the RMET. It is also noted that recent review articles have questioned the psychometric properties of the EQ^79^ and RMET^80^.

The finding that the within-couple correlations for AQ, SQ-R, RMET and EFT were not moderated by length of relationship or social homogamy variables suggests that these effects reflect initial and active assortment (i.e., people seek out similar individuals as partners and do not become more alike over the course of their relationship). Although Pollman et al.^48^ reported that autistic traits were not associated with relationship satisfaction in wives, husbands with high levels of autistic traits had lower relationship satisfaction and this effect was fully mediated by low trust in and responsiveness to their partner as well as low intimacy in the relationship. Perhaps somewhat counterintuitive though is the observation of Jobe and Williams White^81^ that autistic traits are positively correlated with romantic relationship length, though this might be explained by those with high levels of autistic traits typically being resistant to change and so less likely to choose to end a relationship. However, although speculative, this resistance to change might also result in couples being less likely to converge on these measures over the course of their relationship, an idea which, if true, would provide further support for the presence of initial assortment.

It is noted that the within-couple correlations observed for autism-related variables in the current study were only statistically significant in unmarried couples. This was unexpected because couples that have been together for only a relatively short time (< six months)^82^ tend to exhibit couple-similarity effects that are broadly comparable to those observed in more established relationships^24^. It has also been suggested that partner resemblance in married and unmarried couples is ‘overwhelmingly similar’, and, if anything, that married couples tend to be more alike than cohabiting (but unmarried) couples^24^. The sample of married/engaged couples (n = 45) in the current study was slightly smaller than that of unmarried couples (n = 59), and both analyses are likely to be underpowered. This is evidenced by our a priori power calculation indicating that at least n = 67 couples would be required (and for a one-tailed statistical test, whereas we used a two-tailed approach here). Studies utilising larger samples of married and unmarried couples will be required for the nature of these effects to be fully understood.

It is relevant to note that similarity in people’s levels of autistic traits extends beyond romantic relationships, as the phenomenon has also been observed in friendship dyads. Wainer et al.^35^ reported that autistic traits (as measured by the Broad Autism Phenotype Questionnaire [BAPQ]) were significantly correlated within same-sex friendship pairs, and that this effect was present when examined for both self-report (*r* = 0.23) and informant- (i.e., partner) report measures (*r* = 0.16). Of particular relevance here is the finding that concordance on autism-related traits (specifically the ‘aloofness’ scale of the BAPQ) predicted increased relationship satisfaction in newly-formed college roommate dyads when measured at 9–10-week follow-up^83^. Furthermore, autistic adults appear to be more comfortable during first interactions with other autistic (as opposed to neurotypical) adults^77,84^. Taken together, these findings imply that individuals with high levels of autistic traits find it easier to begin relationships with people who show concordance in this regard, and that such relationships are more likely to progress. This process may therefore also be implicated in the development of romantic relationships, with individuals more closely matched on autistic traits being more likely than discordant dyads to pursue and maintain them. However, it is also worth noting that Jobe and Williams White^81^ observed that higher social motivation was correlated with longer friendships, but only in those with an AQ score below the 75^th^ percentile. This might suggest that associations between autistic traits and certain variables relevant to social and/or romantic relationships may not always be linear in nature, and that there may be thresholds above or below which such associations cannot be detected.

There are several limitations to the current research. First, the average level of educational attainment was fairly high (17.6% had a Master’s degree or higher); although arguably more representative than many studies in this area, it is still questionable exactly how generalisable our sample is in regard to the general population. Our study is also correlational in nature, meaning that it was not possible to assess the development of relationships over time to determine causality^31^. Another limitation is that although several researchers gathered data, in the lab study both members of each couple were tested by the same researcher. This could create opportunities for researcher bias and an artificial inflation of couple similarity scores.

Due to COVID-19-related restrictions, only a subsample of our study participants was administered the RMET and EFT, meaning that analyses relating to these variables achieved lower statistical power than those for the AQ, EQ, SQ-R, and D. Although we still demonstrated statistically significant effects relating to the RMET and EFT, replication and extension of these findings will be necessary for firmer conclusions to be drawn. For instance, future studies will be required to determine whether assortative mating processes apply specifically to these variables or whether such effects are explained by within-couple similarity for intelligence quotient (IQ) scores. We also acknowledge that, although it provides a useful indication of effect size for the within-couple correlation for autistic traits, our meta-analysis is limited in certain ways. For instance, due to its ad hoc nature (it was not part of our pre-registration plan), it falls short of PRISMA guidelines^85^, e.g., because we did not conduct systematic literature searches or assess study quality.

In conclusion, the current study provides evidence that small-to-moderate levels of partner similarity likely exist for a range of traits associated with autism spectrum conditions. Although limitations of the analyses conducted should be considered, the observed effects appear more consistent with initial and active assortment than with convergence and social homogamy. Notably, we also demonstrate that systemizing is positively correlated between heterosexual partners, and that actual couples are more similar for this trait than would be expected under the assumption of a random pattern of mating.

## Methods

### Participants

We conducted an a priori power analysis using G*Power 3.1^86,87^ to determine the sample size. Assuming a medium effect size (*r* = 0.30^68^) for within-couple correlations on personality variables (e.g., Kardum et al.^29^) and 80% power, this analysis determined that a sample size of n = 67 couples would be required to observe a statistically significant effect (*p* < 0.05) with a one-tailed Pearson’s correlation test. Adult participants (≥ 18 years) from the UK who were in heterosexual relationships were then recruited from researchers’ contacts and via snowball sampling. Ethical approval was obtained from the Department of Psychology Research Ethics Committee, Birmingham City University (approval number: 172.17), informed consent was provided by all participants, and the procedures were conducted in accordance with the Declaration of Helsinki.

### Apparatus/materials

#### Demographics

Participants first reported their sex (Male, Female, Prefer not to say, Other [please specify below]), age (years), and ethnicity (White, Mixed / multiple ethnic groups, Asian / Asian British, Black / African / Caribbean / Black British, Other ethnic group). They were then asked questions regarding their relationship, specifically their cohabiting status (Living with partner, Not living with partner), length of relationship (years and months), and marital status (Not married, Engaged to be married, Married). They were also asked to confirm their educational level (No qualifications, Completed GCSE level [or equivalent], Completed A level, Access Course [or equivalent], Bachelor’s Degree, Master’s Degree, Doctorate Degree, Other, please specify), current student status (Yes, No; if Yes, then area and year of study were also recorded), whether they were employed (Yes, No; if Yes, then place of work and job role were also recorded), whether they had an autism diagnosis (Yes, No) or suspected they were autistic (Yes, No).

#### Autistic traits and related measures

We used the 50-item self-report Autism Spectrum Quotient (AQ^12^) to measure autistic traits. For each item, participants are asked to specify to what extent they consider a statement to relate to them (response options: Strongly agree, Slightly agree, Slightly disagree, Strongly disagree). Approximately half of the items are reverse-coded, and one point is given for each response (either slight or strong) that indicates an autistic trait. The sum of all 50 items (possible range = 0–50) is calculated as an indicator of one’s level of autistic traits (higher scores signify more autistic traits). The AQ was chosen because it is a well-established measure that has been used extensively in the literature^13^, and because it demonstrates high test–retest reliability (*r* = 0.70 over two-weeks^12^) and construct validity^12,14,88^. Cronbach’s alpha was considered satisfactory (i.e., > 0.70^89^; see also^90^) in the current study (α = 0.823).

We measured self-reported empathizing via the 40-item Empathy Quotient (EQ^66^). Although the response options are the same as those of the AQ, in this case, participants are assigned 1 point for each response that slightly endorses an empathizing tendency and 2 points for a response that strongly endorses an empathizing tendency. Approximately half of the items are reverse-scored, and the possible range of scores is 0–80 (higher scores indicate higher empathizing). Internal consistency for this measure was satisfactory (α = 0.923). Self-reported systemizing was measured via the 75-item Systemizing Quotient-Revised (SQ-R^67^). As with the EQ, 1 point is assigned for each response slightly endorsing a systemizing tendency and 2 points are assigned for each response strongly endorsing a systemizing tendency; scores can range from 0 to 150, and higher scores indicate higher systemizing. The SQ-R showed satisfactory internal consistency in the current study (α = 0.911). In addition to examining EQ and SQ-R, we standardised these scores (in males and females separately) as E and S, respectively, and calculated the difference as: D = S − E^67,91^. D scores provide an indication of one’s cognitive style, with positive values indicating relatively strong systemizing compared to empathizing.

In addition to the self-report questionnaires, two behavioural measures were administered. First, the Reading the Mind in the Eyes Test (RMET^61^) was used to provide an indication of participants’ ability to correctly infer mental states in others, a skill that broadly maps onto empathizing. The RMET is also sometimes thought of as a measure of theory of mind via facial expressions. For this task, a picture of the eye region is shown along with four adjectives, one of which correctly describes the emotion portrayed; a practice trial is completed before the 36 items that comprise the measure. Internal consistency was satisfactory (α = 0.709). We also used the Embedded Figures Test^63^ as a behavioural task that taps into abilities that may be prerequisite to systemizing. For this measure, participants are shown a rectangular stimulus consisting of horizontal, vertical, and diagonal lines, within which they are tasked with identifying a particular shape (i.e., the embedded figure). A practice trial is conducted prior to the 12 trials that comprise the measure. Participants are timed on each trial by a Research Assistant using a stopwatch, and the time taken to identify the correct shape is recorded (if the participant does not identify the correct shape within 180 s, the task proceeds to the next trial). The mean completion time across all 12 trials is computed as the variable of interest. The internal consistency for this measure was satisfactory (α = 0.787).

### Design and procedure

The current study utilised a correlational design. Participants were invited to attend a lab session in which they and their partner would independently complete several measures relating to autism. Each couple was offered a £10.00 shopping voucher as an incentive to participate. The study protocol, hypotheses, and analysis plan were pre-registered on the Open Science Framework (osf.io/6jg8p). (Please note that the pre-registration was put in place after data collection had been completed but before data analysis began.) However, due to restrictions imposed by the Coronavirus Disease (COVID-19) pandemic, a minority of participants completed the study via an online survey hosted by Qualtrics. Digit ratio (2D:4D) was also measured for those participants that attended the lab, and results from that aspect of the study have been published elsewhere^92^.

### Statistical analyses

We examined intercorrelations between the outcome measures via Pearson’s correlations, and tested for sex differences in the autism-related variables by using independent samples *t*-tests. We then examined within-couple associations for age (Pearson’s correlation), educational attainment (Spearman’s correlation) and STEM study/occupational status (chi-square test). These were essentially used as control checks, as positive correlations for age, educational attainment, and occupation are already widely established^31^. It was also important to determine that there were significant correlations for these variables because if there were not, it would not have made sense to examine them to test for evidence of social homogamy (see below).

We conducted variable-centred and couple-centred analyses^93^ to examine within-couple correlations and similarity for each of the autism-related variables. For the variable-centred analysis we used Spearman’s tests to determine the strength and direction of the within-couple correlations. We next used linear regression analyses (controlling for marital status [unmarried or married/engaged]) to determine the level of association between length of relationship and the within-couple difference scores for autism-related variables. The idea behind this analysis was that a significant negative correlation could imply that couples’ scores become more similar over time and so indicate a convergence effect rather than initial assortment. We then performed similar analyses in relation to within-couple differences for age and educational attainment with the idea that a significant positive correlation would indicate that couple similarity for autism-related variables is explainable in terms of social homogamy rather than active assortment. Finally, we used independent samples *t*-tests to determine whether couples for whom both members worked/studied in STEM areas were more similar in terms of autism-related variables than couples for whom only one or neither member worked/studied in STEM.

For the couple-centred analysis we calculated sex-standardised difference scores for autism-related variables for actual couples and used paired samples *t*-tests to compare these with average difference scores calculated by pairing each male in the dataset with each female other than his partner (and, hence, each female was also paired with each male who was not her partner). Whereas the variable-centred analysis essentially determines whether a high or low score in one person is a predictor of whether their partner will have a high or low score on the same measure, the couple-centred analysis can determine whether actual couples’ scores are more alike than expected assuming a pattern of random mating.

Although not specified in our pre-registration document, we conducted a meta-analysis to provide a more precise effect size estimate for the within-couple correlation for autistic traits. We performed a random effects meta-analysis using the R package metafor^94^ so as to allow for the possibility of the true effect size for the correlation differing depending on moderating factors. Due to Pearson’s *r* being non-normally distributed, we converted *r* to Fisher’s *z*, performed the meta-analysis on *z*, and then back-calculated to *r* for ease of interpretation^95^.

We considered *p* < 0.05 (two-tailed) to indicate statistical significance and interpret effect sizes in accordance with Cohen^68^. Data analysis was conducted in R Studio version 4.0.2.

## Data Availability

The datasets supporting the conclusions of this article are available in the Open Science Framework repository [https://osf.io/6jg8p/]^97^.
